# Comparative In Vitro Activity of Ceftazidime-Avibactam, Imipenem-Relebactam, and Meropenem-Vaborbactam against Carbapenem-Resistant Clinical Isolates of *Klebsiella pneumoniae* and *Pseudomonas aeruginosa*

**DOI:** 10.3390/antibiotics13050416

**Published:** 2024-05-01

**Authors:** Anthony Sophonsri, Michelle Kalu, Annie Wong-Beringer

**Affiliations:** Department of Clinical Pharmacy, Alfred E. Mann School of Pharmacy and Pharmaceutical Sciences, University of Southern California, Los Angeles, CA 90089, USA; sophonsr@usc.edu (A.S.); mkalu@usc.edu (M.K.)

**Keywords:** carbapenem resistance, carbapenemase, multidrug-resistant organism, *Pseudomonas aeruginosa*, *Klebsiella pneumoniae*, KPC, VIM, avibactam, relebactam, vaborbactam

## Abstract

Co-infection with carbapenem-resistant *Klebsiella pneumoniae* (CRKP) and *Pseudomonas aeruginosa* (CRPA) is associated with poor outcomes and historically relied on combination therapy with toxic agents for management. However, several novel β-lactam/β-lactamase inhibitor combination agents have been developed, offering potential monotherapy options. Here, we compare the in vitro activity of ceftazidime-avibactam (CZA), imipenem-relebactam (IRL), and meropenem-vaborbactam (MVB) against both CRKP and CRPA clinical isolates. Minimum inhibitory concentrations (MICs) for each agent were determined using broth microdilution. Carbapenemase gene detection was performed for representative isolates of varying carbapenem resistance phenotypes. IRL demonstrated excellent activity against CRKP and CRPA with susceptibility rates at 95.8% and 91.7%, respectively. While CZA and MVB showed comparable susceptibility to IRL against CRKP (93.8%), susceptibility of CRPA to CZA was modest at 79.2%, whereas most CRPA strains were resistant to MVB. Of the 35 CRKP isolates tested, 91.4% (32/35) carried a *bla*_KPC_ gene. Only 1 of 37 (2.7%) CRPA isolates tested carried a *bla*_VIM_ gene, which conferred phenotypic resistance to all three agents. None of the CRKP strains were cross-resistant to all three agents. Source of infection and co-infection did not significantly influence antimicrobial activity for IRL and CZA; none of the CRPA isolates from co-infected patients were susceptible to MVB. Our results suggest that novel β-lactam agents with antipseudomonal activity and stability against carbapenemases, such as IRL and CZA, offer potential monotherapy options for the treatment of co-infection involving both CRKP and CRPA, but not MVB.

## 1. Introduction

Inappropriate prescribing and overuse of antibiotics over the past decades has led to the development and spread of multi-drug resistant organisms (MDROs) worldwide. Compared to infections caused by susceptible bacteria, those caused by MDROs are associated with higher mortality risk and impose a significant economic burden costing over USD 20 billion per year in the United States alone [1,2,3]. According to one study, antimicrobial resistance (AMR) was associated with nearly 5 million deaths worldwide in 2019, of which almost 1.3 million deaths were directly due to bacterial AMR [4]. Despite a global effort to combat antibiotic resistance, the rate at which resistance spreads seemingly outpaces that of effective antimicrobial development. Thus, AMR has become an urgent global public health threat, and poses a significant challenge in patient care as effective treatment options remain limited.

Both carbapenem-resistant *Klebsiella pneumoniae* (CRKP) and *Pseudomonas aeruginosa* (CRPA) are major contributors to antibiotic resistance worldwide and rank among the top three critical MDR pathogens on the World Health Organization’s (WHO) priority list [5]. Adding to the therapeutic challenge is the increasing incidence of co-infection with both carbapenem-resistant pathogens (CRKP and CRPA) necessitating the use of nephrotoxic agents such as colistin in combination therapy or multiple changes in treatment regimens due to suboptimal activity [6,7,8]. We have previously reported that co-infection with CRKP and CRPA is associated with significant morbidity and healthcare burden, underscoring the need for effective empiric therapy at the onset of infection [9]. In the past decade, several novel β-lactam/β-lactamase inhibitor combination agents with antipseudomonal activity and stability against the KPC carbapenemases have become available, making it possible to use monotherapy to treat co-infections involving these pathogens. Therefore, our goal is to compare the in vitro activity of ceftazidime-avibactam (CZA), imipenem-relebactam (IRL), and meropenem-vaborbactam (MVB) against CRKP and CRPA clinical isolates obtained from patients with or without co-infection.

## 2. Results

**Figure 1:**
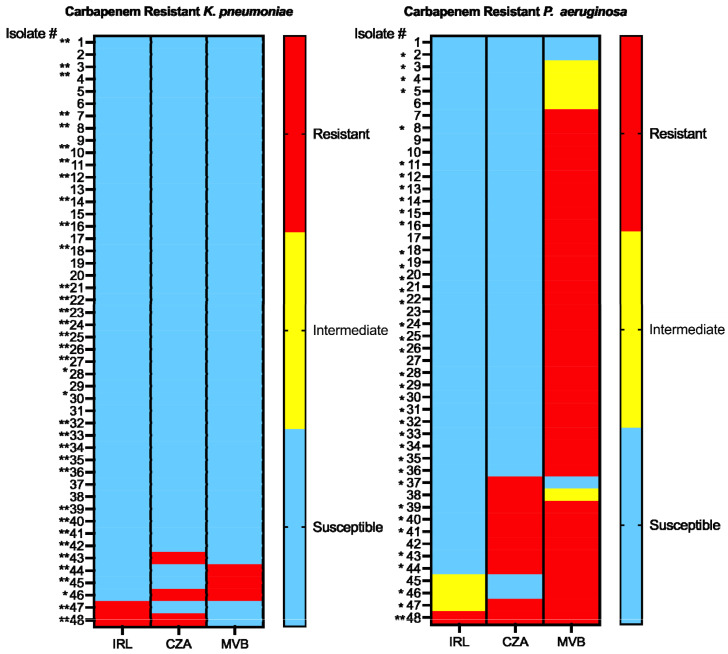
Carbapenem-resistant *K. pneumoniae* (CRKP) and *P. aeruginosa* (CRPA): carbapenem resistant phenotypes and cross-resistance to β-lactam/β-lactamase inhibitor. Resistance breakpoints were defined according to CLSI interpretative criteria [10]. *K. pneumoniae* MIC breakpoints (µg/mL): IRL (S, ≤1/4; I, 2/4; R, ≥4/4), CZA (S, ≤8/4; R, ≥16/4), MVB (S, ≤4/8; I, 8/8; R, ≥16/8). *P. aeruginosa* MIC breakpoints (µg/mL): IRL (S, ≤2/4; I, 4/4; R, ≥8/4), CZA (S, ≤8/4; R, ≥16/4), MVB based on meropenem breakpoints (S, ≤2/8; I, 4/8; R, ≥8/8). Isolates are numbered from 1-48. An asterisk (*) next to the isolate number denotes isolates which tested negative for detectable carbapenemase genes whereas a double asterisk (**) denotes isolates which tested positive for *bla*_KPC_ among *K. pneumoniae* (n = 32/35) or *bla*_VIM_ among *P. aeruginosa* (n = 1/37). IRL, imipenem-relebactam; CZA, ceftazidime-avibactam; MVB, meropenem-vaborbactam.

A total of 96 isolates (CRKP, n = 48; CRPA, n = 48) from both co-infected and mono-infected patients in a non-outbreak setting were tested. The most common infectious sources were the lower respiratory (60.4%, 58/96) and urinary (24%, 23/96) tracts, followed by wound (10.4%, 10/96) and blood (5.2%, 5/96). All isolates remained highly susceptible to all three combination agents tested, except for significant resistance to MVB among CRPA isolates (Table 1 and Figure 1).

Activity against CRKP was similar among the three agents, with IRL (95.8%, 46/48) being the most active. Of the three CRKP isolates resistant (R) to CZA, one was cross-resistant to IRL and one was cross-resistant to MVB. The other CRKP isolate resistant to IRL remained susceptible (S) to both CZA and MVB. The remaining two CRKP isolates resistant to MVB were both susceptible to CZA and IRL. None of the CRKP strains tested were resistant to all three agents. Generally, there were only modest differences in phenotypic resistance between CRKP isolates from co-infected and mono-infected patients. CRKP isolates from co-infected patients were more susceptible to IRL (100% vs. 91.7%) and CZA (100% vs. 87.5%), but were equally susceptible to MVB (91.7% vs. 95.8%) as compared to isolates from mono-infected patients. The source of infection did not have an effect on susceptibility rates.

Similarly, IRL demonstrated the highest activity against CRPA with an overall susceptibility of 91.7% (44/48), followed by CZA at 79.2% (38/48). Notably, CRPA isolates were highly resistant to MVB with only 6.3% (3/48) susceptibility based on meropenem MIC breakpoints. Among the MVB-non-susceptible (NS) isolates, most remained susceptible to IRL (91.1%, 41/45) followed by CZA (80%, 36/45). Of the four IRL-NS CRPA isolates (three resistant, one intermediate), all were cross-resistant to MVB and two (50%) were cross-resistant to CZA. Of the remaining eight CZA-R CRPA isolates, all were susceptible to IRL, whereas only one (12.5%) was susceptible to MVB. Generally, there were only modest differences in phenotypic resistance between CRPA isolates from co-infected and mono-infected patients. CRPA isolates from co-infected patients were more susceptible to IRL (95.8% vs. 87.5%), but were equally susceptible to CZA (79.2%). Notably, CRPA isolates from co-infected patients were less susceptible to MVB (0% vs. 12.5%). The source of infection did not have an effect on susceptibility rates.

A subset of 35 CRKP (single isolation, n = 15; co-isolation, n = 20) and 37 CRPA (single isolation, n = 14; co-isolation, n = 23) isolates representative of varying resistant phenotypes based on MIC to each drug were selected for CARBA-R testing to detect carbapenemase resistance genes (Figure 1). Among the 35 CRKP isolates tested, 91.4% (32/35) carried a *bla*_KPC_ gene. All (20/20) CRKP isolates from co-infected patients carried the *bla*_KPC_ gene while only 80% (12/15) from mono-infected patients were carriers. Among the 37 CRPA isolates tested, only 2.7% (1/37) of CRPA carried a *bla*_VIM_ gene; the latter strain was isolated from a co-infected patient and was resistant to all three β-lactam/β-lactamase inhibitor combination agents tested. Other detectable carbapenemase genes (*bla*_OXA-48_, *bla*_NDM_, and *bla*_VIM_) were not identified in any of the isolates tested.

## 3. Discussion

The increasing incidence of co-infection with both carbapenem-resistant *K. pneumoniae* and *P. aeruginosa* not only imposed significant morbidity and healthcare burden, but also presented a significant therapeutic challenge for clinicians. Historically, co-infection with multi-drug resistant organisms such as CRKP and CRPA necessitated the use of toxic agents such as colistin as part of combination therapy. Our study demonstrates that both imipenem-relebactam (IRL) and ceftazidime-avibactam (CZA) retained high activity against CRKP and CRPA, whereas meropenem-vaborbactam (MVB) demonstrated reliable activity against only CRKP and was mostly non-susceptible against CRPA.

As the majority of tested CRKP isolates harbored the *bla*_KPC_ gene, in vitro efficacy demonstrated by all three agents could be attributed to their respective β-lactamase inhibitor components that confer stability against KPC enzymes. Interestingly, two KPC-producing CRKP isolates were resistant to CZA, but susceptible to MVB. Several studies have demonstrated exposure to CZA led to point mutations in *bla*_KPC-2_ or *bla*_KPC-3_ that modulate KPC Ω-loop stability, which increased ceftazidime affinity conferring CZA resistance and restoring activity to carbapenems [11,12,13,14]. Although IRL retained high activity against CRPA, there were four (8.3%) non-susceptible isolates (three intermediate, one resistant). The resistant isolate harbored the *bla*_VIM_ gene and had an IRL MIC of 16/4 µg/mL while the intermediately susceptible isolates did not have a detectable carbapenemase gene, but had an IRL MIC of 4/4 µg/mL. Production of VIM carbapenemase was likely the primary resistance mechanism against all three agents in the IRL-R CRPA isolate. The mechanism for the increased MIC in the intermediately susceptible isolates remains unclear as IRL is reportedly stable against porin mutations and hyperexpression of efflux pumps which are known to be upregulated among multi-drug resistant *P. aeruginosa* [15,16]. Notably, both MVB and CZA were less effective against CRPA isolates than IRL. Interestingly, MVB susceptibility was significantly lower than CZA (6 % vs. 79%) against CRPA despite the lack of detectable carbapenemase genes. Resistance to CZA and MVB among CRPA is likely multifactorial and could include a combination of increased efflux, reduced membrane permeability, and production of ESBLs [17]. Given that *P. aeruginosa* harbors four major efflux systems (MexAB-OprM, MexXY, MexCD-OprJ, and MexEF-OprN), the discordant phenotypic resistance against CZA and MVB may be due to differential efflux pump-mediated drug extrusion as meropenem is a known substrate of multiple systems (MexAB-OprM, MexCD-OprJ, and MexXY-OprM), whereas ceftazidime is primarily a substrate of MexAB-OprM [15].

Given the potential for horizontal gene transfer (HGT) of antimicrobial resistance mechanisms and virulence factors among co-infected patients, co-isolates were expected to be more resistant [18]. However, susceptibility rates for IRL and CZA did not significantly differ for both CRKP and CRPA isolates from patients with mono- or co-infection; none of the CRPA isolates from co-infected patients were susceptible to MVB compared to 12.5% from mono-infected patients. There were also no CRPA isolates with carbapenemase gene testing that harbored a detectable *bla*_KPC_ gene. Additionally, the CRKP isolate paired with the VIM-producing CRPA isolate harbored a *bla*_KPC_ gene and not the *bla*_VIM_ gene. Despite the potential for HGT among co-infected patients, successful HGT is largely influenced by various factors including environmental conditions as well as alterations in bacterial fitness, intrinsic protein disorder, and gene dosage sensitivity [19]. Although there exists other transferrable resistance mechanisms like those affecting drug transport (e.g., efflux systems and porins) [20,21,22], the lack of any major differences in phenotypic resistance may represent the absence of effective acquisition or expression of any transferrable resistance genes between co-isolates.

We acknowledge that the lack of global isolates may preclude broad generalizability of our results. However, isolates were collected from hospitalized patients in a non-outbreak setting over a span of 11 years from 2012–2022. While not all isolates with phenotypic carbapenem resistance were tested for the presence of carbapenemase genes, we included isolates that represented varying phenotypic resistance to the combination agents. As our focus was to evaluate monotherapy options for co-infection due to CRKP and CRPA with novel β-lactam/β-lactamase inhibitor combination agents, mechanisms of resistance other than the presence of carbapenemase genes were not investigated.

In conclusion, our study found that imipenem-relebactam and ceftazidime-avibactam were highly effective in vitro against both CRKP and CRPA. While similarly effective against CRKP, meropenem-vaborbactam demonstrated very low activity against CRPA. Our results suggest that novel β-lactam agents with antipseudomonal activity and stability against carbapenemases, such as IRL and CZA, offer potential monotherapy options for the treatment of co-infection involving both CRKP and CRPA, obviating clinicians’ dependence on the use of older more toxic agents in combination regimens.

## 4. Materials and Methods

### 4.1. Bacterial Strains

A total of 48 carbapenem-resistant *Klebsiella pneumoniae* (CRKP) and 48 carbapenem-resistant *Pseudomonas aeruginosa* (CRPA) isolates collected from 24 co-infected (2 isolates per patient) and 48 mono-infected patients hospitalized during a span of 11-year period between 2012–2022 were available for this study. Co-infection was defined as bacterial isolation of CRKP and CRPA within the same specimen for a single polymicrobial culture or in separate specimens less than 7 days apart in which CRPA and CRKP isolation occurred on two different cultures. In total, 24 paired isolates of CRKP (n = 24) and CRPA (n = 24) were collected from co-infected patients; the remaining 48 isolates (CRKP, n = 24; CRPA, n = 24) were from mono-infected patients. Isolate identification and susceptibility, used to determine carabapenem resistance, were performed with clinical microbiology using a matrix-assisted laser desorption/ionization-time of flight (MALDI-TOF) mass spectrometry (bioMérieux, Marcy-l’Étoile, France) and a BD Pheonix^TM^ system (Becton, Dickinson and Co., Franklin Lakes, NJ, USA), respectively.

### 4.2. Antimicrobial Susceptibility Testing

Bacterial isolates were cryopreserved in 50% glycerol/50% cation-adjusted Mueller–Hinton broth (CAMHB) at −80 °C. Bacterial cultures were first streaked onto non-selective chromogenic agar plates and selective Pseudomonas isolation agar (PIA) plates from storage for CRKP and CRPA isolates, respectively, then incubated overnight in CO_2_ at 37 °C. After confirmation of growth on chromogenic or selective media, isolates were sub-cultured on tryptic soy agar (TSA) plates and grown in CAMHB overnight. The following antibiotics were tested at a concentration range of 0.0625 µg/mL to 16 µg/mL in 2-fold dilution: ceftazidime (Sigma-Aldrich, Burlington, MA, USA) with 4 µg/mL avibactam (MedChemExpress, Monmouth Junction, NJ, USA), imipenem (Merck & Co., Inc., Rahway, NJ, USA) with 4 µg/mL relebactam (Merck & Co., Inc.), and meropenem (Sigma-Aldrich) with 8 µg/mL vaborbactam (MedChemExpress). Minimum inhibitory concentrations (MICs) were determined using broth microdilution per CLSI standards [10]. Bacterial stock solutions were standardized to ~1 *×* 10^6^ CFU/mL and 50 µL aliquoted into each well of a 96-well clear flat bottom plate (Corning Inc., Corning, NY, USA) that also contained 50 µL of the desired drug concentrations to yield a final inoculum of ~5 *×* 10^5^ CFU/mL per well. Each bacterial isolate was tested in triplicate for each drug combination concentration. Test plates were incubated in CO_2_ at 37 °C overnight for 16–20 h. MICs were determined the following day by visual inspection and confirmed via spectrophotometric analysis at OD_600_ using a Tecan Sunrise absorbance microplate reader (Tecan Group Ltd., Mannedorf, Switzerland).

### 4.3. Carbapenemase Gene Detection

The Xpert^®^ Carba-R assay (Cepheid, Sunnyvale, CA, USA) was used, following the manufacturer’s protocol, to detect and differentiate *bla*_KPC_, *bla*_NDM_, *bla*_VIM_, *bla*_OXA-48_, and *bla*_IMP_ gene sequences encoding for carbapenemases associated with carbapenem non-susceptibility in selected CRKP and CRPA isolates representative of varying carbapenem resistance phenotypes based on MIC (Figure 1).

## Figures and Tables

**Table 1:** Antimicrobial susceptibility results of ceftazidime-avibactam, imipenem-relebactam, and meropenem-vaborbactam against clinical isolates of carbapenem-resistant *K. pneumoniae* and *P. aeruginosa*.

		Overall (n = 48)	*K. pneumoniae* Single Isolation (n = 24)	Co-Isolation (n = 24)	Overall (n = 48)	*P. aeruginosa* Single Isolation (n = 24)	Co-Isolation (n = 24)
Imipenem-relebactam	MIC Range (µg/mL)	≤0.0625, 16	0.125, 16	≤0.0625, 1	0.25, 16	0.25, 4	0.25, 16
	MIC50 (µg/mL)	0.125	0.25	0.125	1	1	1
	MIC90 (µg/mL)	1	1	0.5	2	4	2
	% Susceptible	95.8	91.7	100	91.7	87.5	95.8
Ceftazidime-avibactam	MIC Range (µg/mL)	0.125, >16	0.125, > 16	0.5, 8	0.125, >16	0.125, >16	2, >16
	MIC50 (µg/mL)	2	2	2	8	8	8
	MIC90 (µg/mL)	8	>16	4	>16	>16	16
	% Susceptible	93.8	87.5	100	79.2	79.2	79.2
Meropenem-vaborbactam	MIC Range (µg/mL)	≤0.0625, >16	≤0.0625, >16	≤0.0625, >16	≤0.0625, >16	≤0.0625, >16	4, >16
	MIC50 (µg/mL)	≤0.0625	0.125	≤0.0625	16	16	16
	MIC90 (µg/mL)	4	2	4	>16	>16	>16
	% Susceptible	93.8	95.8	91.7	6.3	12.5	0

NOTE: Single isolation denotes strains isolated as the only identified organism from the culture specimen (mono-infected patients), whereas co-isolation denotes strains where both *P. aeruginosa* or *K. pneumoniae* were grown from the same or discrete cultures within 7 days (co-infected patients). All MIC values represent the β-lactam component only. Concentrations of the β-lactam inhibitor component of all three agents remained constant at 4 µg/mL for avibactam and relebactam, and 8 µg/mL for vaborbactam for every concentration of each combination agent tested. Susceptibility was defined according to CLSI interpretative criteria [10]. *K. pneumoniae*, susceptible MIC breakpoints (µg/mL): IRL (S, ≤1/4;), CZA (S, ≤8/4), MVB (S, ≤4/8). *P. aeruginosa* susceptible MIC breakpoints (µg/mL): IRL (S, ≤2/4), CZA (S, ≤8/4), MVB based on meropenem breakpoints (S, ≤2/8).

## Data Availability

The raw data supporting the conclusions of this article will be made available by the authors on request.

## References

[1] Cosgrove S.E. (2006). The relationship between antimicrobial resistance and patient outcomes: Mortality, length of hospital stay, and health care costs. Clin. Infect. Dis..

[2] Sydnor E.R., Perl T.M. (2011). Hospital epidemiology and infection control in acute-care settings. Clin. Microbiol. Rev..

[3] DiazGranados C.A., Zimmer S.M., Klein M., Jernigan J.A. (2005). Comparison of mortality associated with vancomycin-resistant and vancomycin-susceptible enterococcal bloodstream infections: A meta-analysis. Clin. Infect. Dis..

[4] Wagenlehner F.M.E., Dittmar F. (2022). Re: Global Burden of Bacterial Antimicrobial Resistance in 2019: A Systematic Analysis. Eur. Urol..

[5] WHO Priority Pathogens List for R&D of New Antibiotics. www.who.int/mediacentre/news/releases/2017/bacteria-antibiotics-needed/en/.

[6] Chen Z., Chen Y., Fang Y., Wang X., Chen Y., Qi Q., Huang F., Xiao X. (2015). Meta-analysis of colistin for the treatment of *Acinetobacter baumanii* infection. Sci. Rep..

[7] Doi Y. (2019). Treatment Options for Carbapenem-resistant Gram-negative bacterial infections. Clin. Infect. Dis..

[8] Bassetti M., Echols R., Matsunaga Y., Ariyasu M., Doi Y., Ferrer R., Lodise T.P., Naas T., Niki Y., Paterson D.L. (2021). Efficacy and safety of cefiderocol or best available therapy for the treatment of serious infections caused by carbapenem-resistant Gram-negative bacteria (CREDIBLE-CR): A randomised, open-label, multicentre, pathogen-focused, descriptive, phase 3 trial. Lancet Infect. Dis..

[9] Sophonsri A., Kelsom C., Lou M., Nieberg P., Wong-Beringer A. (2023). Risk factors and outcome associated with coinfection with carbapenem-resistant *Klebsiella pneumoniae* and carbapenem-resistant *Pseudomonas aeruginosa* or *Acinetobacter baumanii*: A descriptive analysis. Front. Cell Infect. Microbiol..

[10] Clinical and Laboratory Standards Institute (2018). Methods for Dilution Antimicrobial Susceptibility Tests for Bacteria That Grow Aerobically.

[11] Gaibani P., Campoli C., Lewis R.E., Volpe S.L., Scaltriti E., Giannella M., Pongolini S., Berlingeri A., Cristini F., Bartoletti M. (2018). In vivo evolution of resistant subpopulations of KPC-producing *Klebsiella pneumoniae* during ceftazidime/avibactam treatment. J. Antimicrob. Chemother..

[12] Giddins M.J., Macesic N., Annavajhala M.K., Stump S., Khan S., McConville T.H., Mehta M., Gomez-Simmonds A., Uhlemann A.C. (2018). Successive Emergence of Ceftazidime-Avibactam Resistance through Distinct Genomic Adaptations in *bla*KPC-2-Harboring *Klebsiella pneumoniae* Sequence Type 307 Isolates. Antimicrob. Agents Chemother..

[13] Shields R.K., Nguyen M.H., Press E.G., Chen L., Kreiswirth B.N., Clancy C.J. (2017). Emergence of ceftazidime-avibactam resistance and restoration of carbapenem susceptibility in *Klebsiella pneumoniae* carbapenemase-producing *K. pneumoniae*: A case report and review of literature. Open Forum Infect. Dis..

[14] Haidar G., Clancy C.J., Shields R.K., Hao B., Cheng S., Nguyen M.H. (2017). Mutations in *bla*KPC-3 that confer ceftazidime-avibactam resistance encode novel KPC-3 variants that function as extended-spectrum β-Lactamases. Antimicrob. Agents Chemother..

[15] Masuda N., Sakagawa E., Ohya S., Gotoh N., Tsujimoto H., Nishino T. (2000). Substrate specificities of MexAB-OprM, MexCD-OprJ, and MexXY-oprM efflux pumps in *Pseudomonas aeruginosa*. Antimicrob. Agents Chemother..

[16] Xavier D.E., Picão R.C., Girardello R., Fehlberg L.C., Gales A.C. (2010). Efflux pumps expression and its association with porin down-regulation and beta-lactamase production among *Pseudomonas aeruginosa* causing bloodstream infections in Brazil. BMC Microbiol..

[17] Flury B.B., Bösch A., Gisler V., Egli A., Seiffert S.N., Nolte O., Findlay J. (2023). Multifactorial resistance mechanisms associated with resistance to ceftazidime-avibactam in clinical *Pseudomonas aeruginosa* isolates from Switzerland. Front. Cell Infect. Microbiol..

[18] Michaelis C., Grohmann E. (2023). Horizontal Gene Transfer of Antibiotic Resistance Genes in Biofilms. Antibiotics.

[19] Acar Kirit H., Lagator M., Bollback J.P. (2020). Experimental determination of evolutionary barriers to horizontal gene transfer. BMC Microbiol..

[20] Ball P.R., Shales S.W., Chopra I. (1980). Plasmid-mediated tetracycline resistance in *Escherichia coli* involves increased efflux of the antibiotic. Biochem. Biophys. Res. Commun..

[21] McMurry L., Petrucci R.E., Levy S.B. (1980). Active efflux of tetracycline encoded by four genetically different tetracycline resistance determinants in *Escherichia coli*. Proc. Natl. Acad. Sci. USA.

[22] Daigle D.M., Cao L., Fraud S., Wilke M.S., Pacey A., Klinoski R., Strynadka N.C., Dean C.R., Poole K. (2007). Protein modulator of multidrug efflux gene expression in *Pseudomonas aeruginosa*. J. Bacteriol..

